# Robust deep learning classification of adamantinomatous craniopharyngioma from limited preoperative radiographic images

**DOI:** 10.1038/s41598-020-73278-8

**Published:** 2020-10-09

**Authors:** Eric W. Prince, Ros Whelan, David M. Mirsky, Nicholas Stence, Susan Staulcup, Paul Klimo, Richard C. E. Anderson, Toba N. Niazi, Gerald Grant, Mark Souweidane, James M. Johnston, Eric M. Jackson, David D. Limbrick, Amy Smith, Annie Drapeau, Joshua J. Chern, Lindsay Kilburn, Kevin Ginn, Robert Naftel, Roy Dudley, Elizabeth Tyler-Kabara, George Jallo, Michael H. Handler, Kenneth Jones, Andrew M. Donson, Nicholas K. Foreman, Todd C. Hankinson

**Affiliations:** 1 Division of Pediatric Neurosurgery, Children’s Hospital Colorado, Aurora, 80045 USA; 2 Department of Neurosurgery, University of Colorado School of Medicine, Aurora, 80045 USA; 3 Morgan Adams Foundation Pediatric Brain Tumor Research Program, Aurora, 80045 USA; 4 Division of Pediatric Radiology, Children’s Hospital Colorado, Aurora, 80045 USA; 5 Department of Neurosurgery, University of Tennessee Health and Sciences Center, Memphis, 38163 USA; 6 Semmes Murphy Clinic, St. Jude Children’s Research Hospital, Memphis, 38105 USA; 7 Neurosurgical Associates of New Jersey, Ridgewood, NJ 07450 USA; 8 Department of Pediatric Neurosurgery, Nicklaus Children’s Hospital, Miami, 33155 USA; 9 Department of Pediatric Neurosurgery, Lucile Packard Children’s Hospital at Stanford University, Palo Alto, 94305 USA; 10 Department of Neurosurgery, Memorial Sloan Kettering Cancer Center, New York, 10065 USA; 11 Department of Neurological Surgery, Weill Cornell Medical College, New York, 10065 USA; 12 Division of Pediatric Neurosurgery, University of Alabama at Birmingham, Birmingham, 35233 USA; 13 Department of Neurosurgery, Johns Hopkins University School of Medicine, Baltimore, 21205 USA; 14 Department of Pediatrics, Washington University School of Medicine, St. Louis, 63110 USA; 15 Department of Pediatric Hematology-Oncology, Arnold Palmer Hospital, Orlando, 32806 USA; 16 Division of Pediatric Neurosurgery, Nationwide Children’s Hospital, Columbus, 43205 USA; 17 Departments of Pediatrics and Neurosurgery, Emory University School of Medicine, Atlanta, 30322 USA; 18 Children’s National Health System, Brain Tumor Institute, Washington, DC 20010 USA; 19 Division of Pediatric Hematology and Oncology, Children’s Mercy Hospital, Kansas City, 64108 USA; 20 Department of Neurological Surgery, Monroe Carell Jr. Children’s Hospital at Vanderbilt, Nashville, 37212 USA; 21 Department of Neurosurgery, McGill University, Montreal, H3A 2B4 Canada; 22 Department of Neurological Surgery, University of Pittsburgh, Pittsburgh, 15213 USA; 23 Institute of Brain Protection Sciences, Johns Hopkins All Children’s Hospital, St Petersburg, 33701 USA; 24 University of Oklahoma Health Sciences Center, Oklahoma City, 73104 USA; 25 Division of Pediatric Neurooncology, Children’s Hospital Colorado, Aurora, 80045 USA

**Keywords:** Paediatric cancer, Machine learning, Cancer imaging

## Abstract

Deep learning (DL) is a widely applied mathematical modeling technique. Classically, DL models utilize large volumes of training data, which are not available in many healthcare contexts. For patients with brain tumors, non-invasive diagnosis would represent a substantial clinical advance, potentially sparing patients from the risks associated with surgical intervention on the brain. Such an approach will depend upon highly accurate models built using the limited datasets that are available. Herein, we present a novel genetic algorithm (GA) that identifies optimal architecture parameters using feature embeddings from state-of-the-art image classification networks to identify the pediatric brain tumor, adamantinomatous craniopharyngioma (ACP). We optimized classification models for preoperative Computed Tomography (CT), Magnetic Resonance Imaging (MRI), and combined CT and MRI datasets with demonstrated test accuracies of 85.3%, 83.3%, and 87.8%, respectively. Notably, our GA improved baseline model performance by up to 38%. This work advances DL and its applications within healthcare by identifying optimized networks in small-scale data contexts. The proposed system is easily implementable and scalable for non-invasive computer-aided diagnosis, even for uncommon diseases.

## Introduction

Deep learning is a subtype of artificial intelligence that constructs generalizable models for data representations via a multilayer abstraction process^1^. A common deep learning architecture used for classification of visual information is known as a Convolutional Neural Network (CNN). CNNs are constructed using multiple sequential layers containing variants of the multi-layer perceptron. These networks have demonstrated generalization capacity for identifying both linear and non-linear latent patterns that lead to differentiable information^2^. CNNs and other variants have had great success in tasks such as image object recognition; speech recognition, translation, and generation; and medical diagnostics, genetics, and drug discovery^3^. These applications have achieved remarkable success, to some extent by leveraging very large amounts of labeled training data. An example is the ImageNet Large Scale Visual Recognition Challenge (ILVSRC). This leading image recognition competition challenges competitors to advance the state of the art in computer-guided object detection and classification. Using the ImageNet dataset, comprising over 1.4 million images across more than 1000 possible categories, CNNs are achieving error rates under 5%^4^.

Within the healthcare space, reliable CNN inference models have been described under conditions when vast amounts of training data are available. Examples include dermatological diseases and diabetic retinopathy^3–5^. However, when such models are trained on more limited datasets, the results are often unreliable, as the models overfit the training data. More specifically, in a small-data context, the latent features that a network models are likely to result from sampling noise that exists only in the training data, and not in novel test data^5^. Without techniques to overcome this generalization problem, CNNs may have limited applications for less common diseases, including brain tumors.

One technique available to overcome the overfitting complication of small training datasets is Transfer Learning (TL). This is a machine learning methodology for storing knowledge gained from solving a problem within one domain and applying that knowledge to another domain^6,7^. The success of TL has led to the development of publicly available pre-trained models derived from top ILSVRC solutions. By using these pre-trained networks to generate feature embeddings for our dataset of interest, we enable our classifier to have access to the pattern recognition capabilities of these state-of-the-art architectures.

Another technique commonly applied to image classification problems is data augmentation. This process synthetically expands a dataset by applying transformations (i.e. crop, rotate, blur, etc.) to real data in an attempt to preserve domain-specific features. We employed two separate data augmentation approaches. The first was a stochastic process that sampled across transformations with probability thresholds. The second method, known as TANDA (Transformation Adversarial Networks for Data Augmentation), is a ML-based approach that uses Generative Adversarial Networks (GANs) and Recurrent Neural Network (RNNs) to learn the optimal combination and parameters of the image transformations within a specific dataset^8^. TANDA was reported to yield synthetic data in which feature representations are distributed and invariant, thus helping disentangle the factors of variation between the two classes^7^.

An additional challenge in identifying the optimal model is the optimization of CNN hyperparameters. This remains a complicated and computationally intensive task^7^. To mitigate the computational time required, one may apply a meta-heuristic parameter optimization in the form of an asynchronously parallelized genetic algorithm. This optimization procedure allows the model to optimize more intelligently over the solution space with fewer required iterations.

To demonstrate the capacity of combining deep networks, transfer learning, data augmentation, and genetic algorithms to overcome the problem of overfitting with small datasets, we utilized the pediatric brain tumor Adamantinomatous Craniopharyngioma (ACP).

ACP is a neurologically devastating brain tumor that is notorious for causing vision loss, hypothalamic injury, hormone dysfunction and cerebrospinal fluid pathway obstruction, among other injuries. This damage results from growth of the tumor in the sellar/suprasellar region of the brain, where it invariably develops. Here, ACP compresses the optic apparatus, hypothalamic-pituitary axis, and cerebral ventricular system. While ACP is a histologically benign lesion, it often recurs locally, which makes further treatment more perilous for the patient. As such, ACP has been associated with the lowest quality of life scores of any pediatric brain tumor^9^. Current therapeutic management of ACP is limited to either aggressive surgical resection or surgical debulking followed by external beam radiation. This differs considerably from the therapy for other tumors that present in the sellar/suprasellar region. For example, Germinoma, one of the most common tumors in the radiographic differential diagnosis of ACP, is effectively treated without surgical intervention. Other masses of this region, including glioma, pituitary adenoma, arachnoid cysts, and others, similarly require therapy tailored to the particular entity. As such, a priori knowledge of the patients diagnosis would considerably improve the clinical care of children with tumors of the sellar/suprasellar region, the most common of which is ACP.

Radiographically, ACP is characterized by heterogeneous solid tissue, cystic regions, and calcification^10^. Recent data indicate that ACP and other tumors of the sellar/suprasellar region may be accurately diagnosed using current radiographic techniques in 64–87%^10^ of cases. This sets a high bar for machine-aided diagnoses, but also leaves room for clinically relevant improvement.

While ACP is the most common sellar/suprasellar pediatric tumor, it is an uncommon entity, representing 2 to 5% of all pediatric brain tumors, with an incidence of approximately 1.9 per million patient-years^10^. In order to facilitate research into this tumor, Advancing Treatment for Pediatric Craniopharyngioma was formed in 2015. This consortium includes 17 North American centers, which share tissue and clinical data regarding children with ACP, thus providing source data for this research. In addition to the imaging data assembled from these centers, we added data from St. Jude Children’s Research Hospital, thereby assembling a generalizable and representative dataset of both ACP and other sellar/suprasellar entities for model training and evaluation.

In summary, ACP is an ideal candidate for CNN inference due to its consistent anatomical location, radiographically recognizable features, and, most importantly, the substantial clinical management differences between ACP and the other brain masses that lie within the differential diagnosis. However, given its incidence, ACP lacks the volume of labeled data observed in more common disease contexts. By describing a mathematical model for the identification of ACP, we present a computationally economical method to optimize CNN architectures for image classification in contexts that do not afford large amounts of labeled training data. In so doing, we create a non-invasive diagnostic tool to aid in the reduction of mis-diagnoses and unnecessary medical intervention.

## Results

### Baseline predictive results

**Figure 1:**
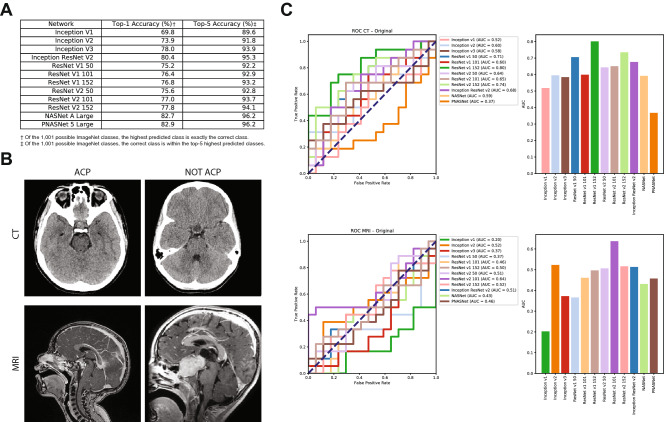
Transfer learning networks, feature embeddings, and baseline results. (**A**) ILSVRC network models utilized, with their top 1% and top 5% accuracy in ILSVRC competition noted. (**B**) Example CT and MRI images for both ACP and NOTACP. (**C**) ROC (left) and AUC (right) values for all twelve networks and both imaging modalities (CT top, MRI bottom). The diagonal dashed line represents performance of a random guess.

Using twelve state-of-the-art networks that have publicly available deep learning models from the TensorFlow Hub library^11^ trained on the ImageNet ILSVRC dataset (Fig. 1A)^4,12–17^, we generated feature embedding vectors to be used in model training (Fig. 1B). Baseline experiments were conducted by training a single fully-connected layer with a softmax activation function and stochastic gradient descent (SGD) optimization algorithm. Using whole-batch training, a learning rate of 0.01, and a training duration of 100 epochs, we established baseline results (Fig. 1C). Across all twelve feature embeddings, on average the classifier accurately labelled individual CT scans 62.3% (Top-5 Network Average $({\hbox {T5NA}}) = 73.3\%$; maximum performance $({\hbox {MP}}) = 80.0\%$) and MRI scans 45.7% (${\hbox {T5NA}} = 54.0\%$; ${\hbox {MP}} = 64.7\%$) of the time.

### Model selection and parameter optimization using manual selection

**Figure 2:**
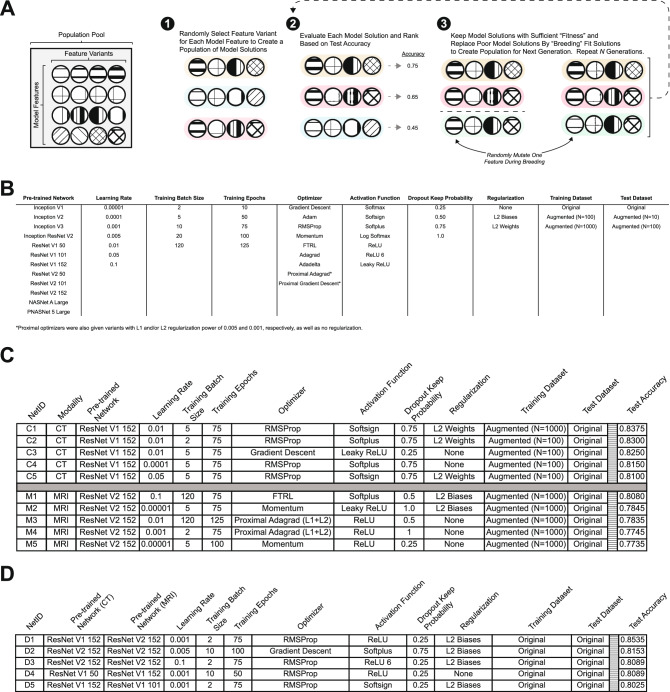
Genetic algorithm optimization of model parameters. (**A**) General process schematic for genetic algorithm parameter optimization. Moving from left to right, a feature variant is selected for each model feature to create individual networks (Step 1; individuals are highlighted in unique colors). Individuals are trained and evaluated to determine fitness and ranked accordingly (Step 2). Two networks are chosen from the fittest population and a new network is derived by selecting from feature variants in these two networks, and variants are occasionally mutated (i.e., randomly selected from the population pool; Step 3). (**B**) Model feature and respective feature variants explored in first phase of genetic algorithm optimization. Each column represents a model feature to be optimized and each row is a possible feature variant for the GA to select from. This table reflects the “Population Pool” (**A**). (**C**) Top-5 performing networks for independent CT and MRI networks after 10 generations of 100 solution populations; ranked according to test accuracy. (**D**) Top-5 performing networks for combined CT-MRI networks after 10 generations of 100 solution populations; ranked according to test accuracy.

To address the computational and time demands associated with architecture selection and hyperparameter optimization within deep learning models^2^, we employed a parallelized simple genetic algorithm (GA) to more rapidly identify optimal combinations of feature extractors, learning parameters, and hyperparameters for both CT and MRI (see Computational Methods; Fig. 2A).

We employed ten different model features to optimize the parameters of the (1) fully connected classifier network; (2) the pre-trained deep CNN to be utilized for feature embeddings; and, (3) the type of training and test datasets (e.g. original or synthetically expanded by augmentation; Fig. 2B). The number of variants for each model feature ranged from three to fifteen, making the total number of possible network combinations 19,051,200 (see "Methods" section). The GA allowed for more intelligent exploration of the solution space and reduced the overall computational time required. By performing 10 generations of 100 solution populations with a generational retention rate of 40%, a negative rejection probability of 10%, and a mutation frequency of 20%, we explored only $1\times 10^3$ solutions to identify the top 5 performing networks of the final generation (Fig. 2C). This process yielded an accuracy increase in CT of only 3.75% (${\hbox {T5NA}} = 82.3\%$; ${\hbox {MP}} = 83.8\%$) and an an increase of 16.8% (${\hbox {T5NA}} = 80.3\%$; ${\hbox {MP} }= 83.3\%$) for MRI.

### Model selection and parameter optimization using a simple genetic algorithm

When the genetic algorithm was employed, a superior network became apparent for both CT- and MRI-trained classifiers. Interestingly, the same general architecture—ResNet—was selected, with CT data favoring the V1 variant and MRI favoring the V2 variant. The primary difference between these architectures is the use of batch normalization (BN) between every layer in V2 as opposed to V1. The BN transformation is particularly resilient toward parameter scale because backpropagation through a layer is unaffected by the scale of its parameters^13^. This suggests that MRI data contained more erratic feature distributions than CT and therefore benefited from the more regularized representation. Further, the superiority of each respective network is highlighted by their selection within the combined CT-MRI network (${\hbox {T5NA}} = 81.7\%; {\hbox {MP}} = 85.4\%$; Fig. 2D).

**Figure 3:**
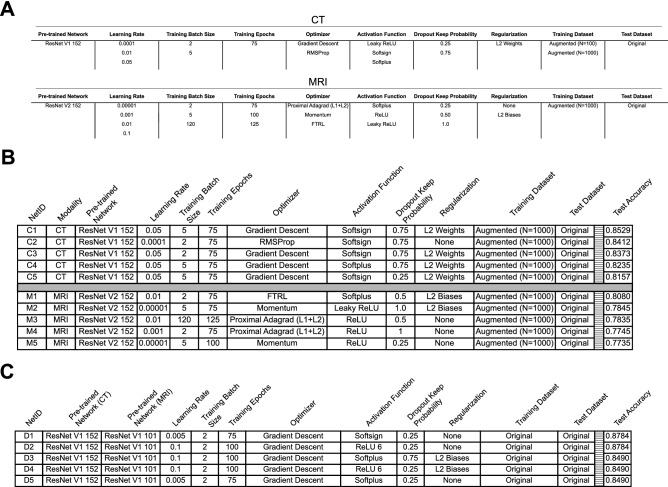
Further optimization with GA. (**A**). Model features and variants available for solution search space in second phase of GA optimization. Each column represents a model feature to be optimized and each row is a possible feature variant for the GA to select from. (**B**) Top-5 performing networks from CT and MRI trained networks as optimized by the GA for 10 generations of 100 solution populations. (**C**) Top-5 performing networks for combined CT-MRI trained networks as optimized by the GA for 10 generations of 100 solution populations.

While performance improved within both modalities compared to the pre-GA results, this method searched only 0.005% of the total number of solutions. We sought to verify that we did not identify a local minima within the model solution space by exploring 0.1% of all solutions. In every GA iteration all paramaters were equally distributed in the first generation, but by the last generation the same end-point was reached with similar results via similar “evolutionary” paths to those presented in Fig. 2 (data not shown). To further improve model generalization, we performed a second iteration of the GA with fewer parameters (Fig. 3A). In this iteration, we allowed the GA to evolve a parameter population with only 144 possible combinations for CT and 1944 possible solutions for MRI. After running the GA on the new refined feature lists for 10 generations with 100 solutions per generation, the top accuracy for CT increased by a further 1.54% (T5NA = 83.4%; MP = 85.3%; Fig. 3B). Similarly, we observed an increase in performance for CT-MRI networks (T5NA = 86.1%; MP = 87.8%; Fig. 3C). Interestingly, however, the solution networks for MRI classification did not attain the same level of accuracy as the initial GA iteration (T5NA = 78.5%; MP = 80.8%; Fig. 3B). This could be due to our optimization algorithm erroneously identifying a local minimum of the solution space, as opposed to the desired global minimum.

### Stochastically augmented training data outperforms TANDA augmented training data

**Figure 4:**
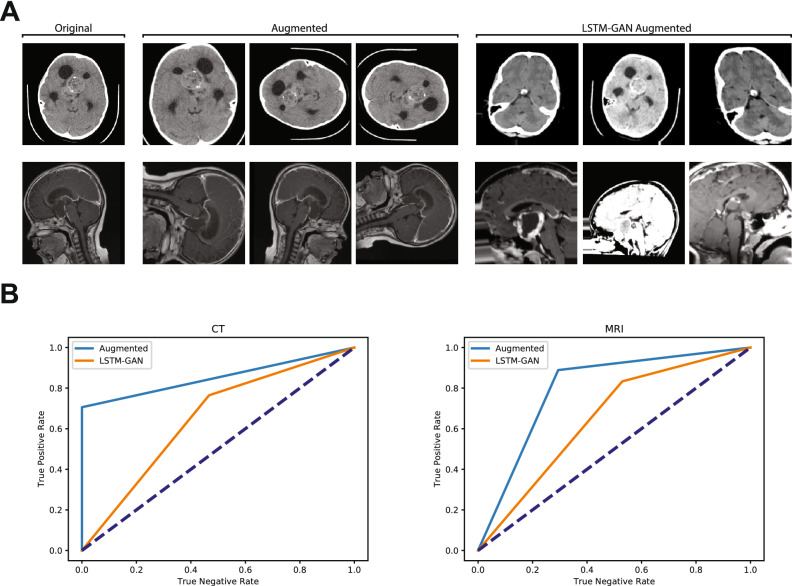
Test performance of models trained on stochastic image augmentation and GAN-LSTM augmented images. (**A**) Exemplar images of original training CT (top) and MRI (bottom), with randomly augmented variants, and TANDA-augmented variants. (**B**) ROC curves for CT- and MRI-trained networks comparing top results of supervised augmented images and TANDA-generated images. Dashed lines represent ROC curve of random chance classification.

It is well documented that data augmentation improves performance of state-of-the-art image classification models^18,19^. Two potential data augmentation solutions are the stochastic-based and ML-based augmentation pipelines. We explored both solutions, using the publicly available python modules Augmentor (stochastic-based) and TANDA (LSTM-GAN-based) to oversample our training data to 1000 images per class (Fig. 4A). Interestingly, optimal models trained on stochastically augmented data outperformed those same architectures trained on TANDA augmented data (Fig. 4B).

This finding could result from our use of TANDA with parameters given for the MNIST handwritten-image dataset context, rather than applying TANDA following optimization for our specific context. While this represents an area for additional investigation, current literature suggests that TANDA can be expected to realize improvements of 2–4% in CNNs with architectures comparable to our own^8^. Given this fairly small improvement, there exists a computational efficiency argument that favors the use of the stochastic process, especially in early stage investigations, or in contexts where computational resources are limited. In our study, the stochastic approach yielded superior results without the requirement to train and evaluate complex machine-learning systems and with lesser computational demand.

An additional aspect of our data that could favor the use of stochastic data augmentation is the relative conspicuity of the critical features of the image. Both due to the nature of a tumor being a mass lesion, and the quality of current medical imaging technologies, the primary source of differentiable information in the images that compose our dataset lies within the sellar/suprasellar region (discussed in the following section), with a gradient of decreasing value as one moves radially away from this region. The resultant relative simplicity in the images may therefore lead to only a marginal difference between stochastic augmentation and TANDA. Datasets in which the target object is more difficult to distinguish from the background (for example, identifying a person wearing black and white stripes among a group of zebras) may, however, better demonstrate the advantage of the more complex TANDA methodology.

### Manual objective obfuscation indicates the sellar/suprasellar region is critical to class identification

**Figure 5:**
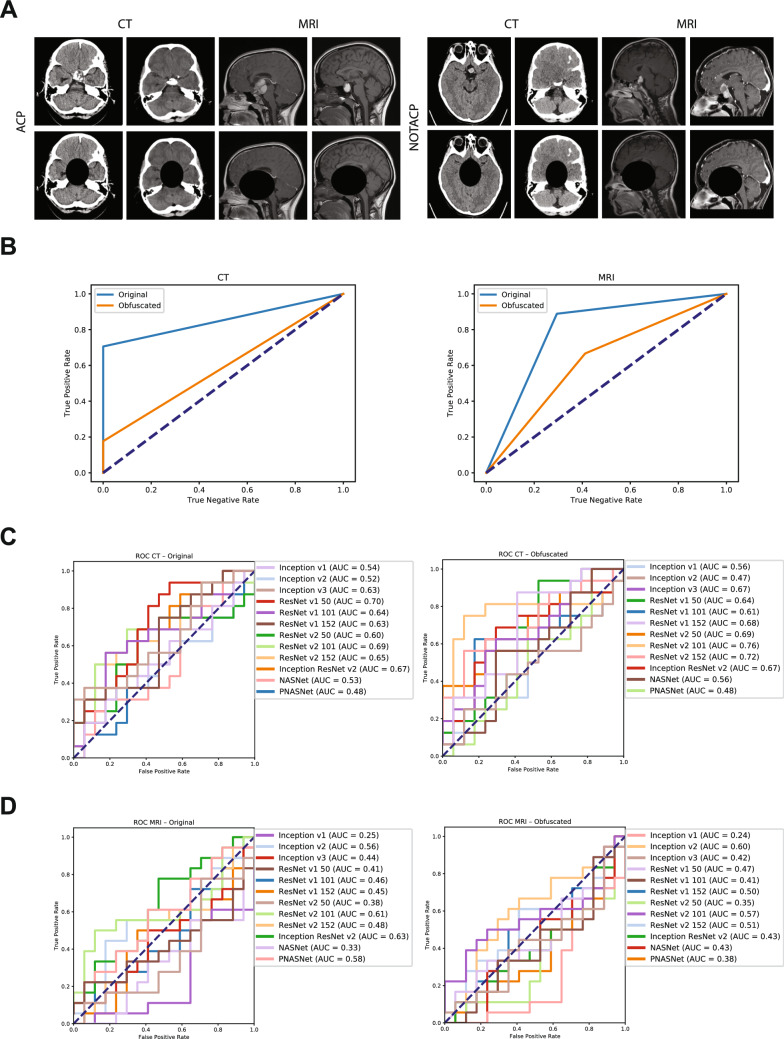
Pituitary obfuscation reveals latent features exist outside canonical ROI for CT scans. (**A**) Example original and obfuscated images for both data classes and both imaging modalities. (**B**) ROC curves for networks trained on obfuscated and original data; original data was ‘Augmented (${\hbox {N}}=1000$)’ variant. (**C**) Baseline ROC curves for all twelve networks trained on original (left) and obfuscated (right) CT images. (**D**) Baseline ROC curves for all twelve networks trained on original (left) and obfuscated (right) MRI images.

To understand the general patterns the model identified as class indicators, we performed manual objective obfuscation of the sellar/suprasellar region in all training images (Fig. 5A). The previously identified optimal networks were trained on these obfuscated data and subsequently used to infer diagnosis from the test set. In this context, the networks failed to accurately distinguish ACP from NOTACP images (Fig. 5B). Interestingly, however, when the baseline networks were trained using obfuscated data, some networks reliably distinguished data classes (Fig. 5C,D). This suggests that while the GA-identified networks utilize image patterns within the sellar/suprasellar region, other non-optimized networks identify latent patterns outside of the sellar/suprasellar region, which is the anatomical location of ACP. As such, a potential improvement to our model could be to integrate feature embeddings from all networks in order to leverage both sellar/suprasellar and extra-sellar patterns within the data.

### Benchmarking against human performance and assessment of hold out training/testing approach

**Figure 6:**
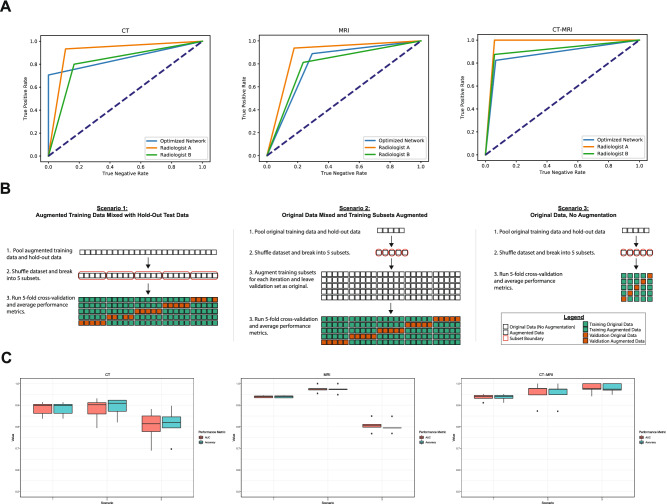
Optimized network classification performance versus human specialist and 5-fold cross-validation evaluation. (**A**) Radiologist average auROC of 89.4%, 83.3%, and 93.8% for CT, MRI, and CT-MRI, respectively. GA-optimzed auROC of 85.3%, 83.3%, and 87.8% for CT, MRI, and CT-MRI, respectively. (**B**) Schematics of 5-fold cross-validation (5F-CV) approaches used to verify the perceived improvement yielded by augmented training data (scenario 3 vs. scenarios 1 and 2). Additionally scenarios 1 and 2 investigate the effect of mixing augmented data into the overall data pool versus only augmenting training data. (**C**) Performance metrics (AUC: area under the ROC curve; Accuracy: standard accuracy metric) for 5F-CV across all three scenarios. Peak performance was achieved via scenario 2 in CT (AUC = 88.0%, Accuracy = 89.0%) and MRI (AUC = 97.5%, Accuracy = 97.4%). In the context of CT-MRI, peak performance was attained in scenario 3 (AUC = 97.8%, Accuracy = 97.9%).

Next, we sought to compare the generalization capacity of our GA-optimized models against the performance of board-certified pediatric neuroradiologists. Using the same test dataset (1 JPEG image per modality per unique patient) used to determine ‘fitness’ within the GA, two specialists were asked to classify diagnosis of ACP/NOTACP in a binary context (Fig. 6). Our optimal models performed on par with the average of human specialists (${p}=0.39$), although ‘Radiologist A’ consistently outperformed our models across the board. As mentioned previously, recent work reported an accuracy of ACP diagnosis of 87% by pediatric neuroradiologists using a complete imaging dataset and clinical history^10^. This performance corroborates the overall generalization capacity of the models presented herein.

Since the dataset utilized is small and therefore sensitive to selection bias, there is concern that the hold-out approach may misrepresent the true predictive capacity of our classifier. To address this, we additionally evaluated our classifier using five-fold cross-validation (5F-CV). Our GA previously identified that augmented data were ideal for training and original data were ideal for test data, thus we chose to evaluate 5F-CV in three different scenarios to verify that augmenting training data are superior (Fig. 6). To further verify the power of our classifier, we chose two separate approaches as to when data is augmented. Interestingly, the 5F-CV data suggests model performance greater than results yielded in the hold-out approach (Fig. 6C). We see that for CT and MRI contexts, augmenting only training data yields the highest results (88.9% and 97.3%, respectively; Fig. 6B. Scenario 2) and classifiers trained on original data perform worse, as expected. This is particularly interesting due to the expectation that mixing augmented data (Fig. 6B. Scenario 1) should increase data overlap and therefore lead to overfitting and inflated performance metrics. Lastly, the CT-MRI context demonstrated that peak performance was attained using original training and test data. Since the CT-MRI context concatenates feature embeddings along a 1-dimensional axis, perhaps these features contain sufficient classification information without the need for augmentation. In fact, information needed in the CT-MRI context may be obscured or dropped by concatenating two augmented case examples. In summary, the combination of hold-out and 5F-CV performance metrics firmly verifies the robustness of our GA-based approach and our resultant classifiers.

## Discussion

Using the pediatric brain tumor Adamantinomatous Craniopharyngioma as an example of a clinical entity with a small available dataset, we enhance the performance of a baseline Convolutional Neural Network using a series of optimization methodologies, including Transfer Learning, Data Augmentation (supervised and unsupervised), and Image Obfuscation. The application of a Genetic Algorithm as a meta-heuristic optimizer realized performance improvements of approximately 23% for CT-trained networks, and 38% for MRI-trained networks, leading to test accuracies of 85.3%, 83.3%, and 87.8% for Computed Tomography (CT), Magnetic Resonance Imaging (MRI), and combined CT and MRI datasets, respectively. We further demonstrate that this is equivalent to the diagnostic accuracy of clinical experts (${p}=0.39$). Lastly, we verified the results of the hold-out test set approach we utilized by demonstrating increased performance under the auspices of 5-fold cross-validation.

Notably, in line with human performance, combining CT and MRI together resulted in higher performance across the board. This is likely due to the increase in relevant information put forth by consideration of both imaging modalities. Furthermore, it is also interesting that we see the baseline performance of CT being very close to the optimized performance in contrast to the larger performance improvement seen in the context of MRI. This is likely due to the intrinsic differences between each classification problem. Meaning, that the pre-trained network feature (as opposed to learning rate, batch size, regularization, etc.) in the CT scenario had the relative greatest impact on overall model performance. Through this kind of perspective it is possible to utilize the GA to extract feature importance information from the GA search space. Additionally, the asynchronous parallelization of our optimization algorithm increased efficiency both in terms of the number of solutions to consider as well as the computational time and resources required to complete calculations. This offers evidence that these techniques may be broadly applied to the development of other parameterized machine learning models in the context of limited training data.

As this work represents an initial exploration of these methodologies, the presented model may be improved. For example, it is possible that the TANDA algorithm could itself be optimized by a GA or other meta-heuristic algorithm, such as particle swarm optimization. Another possible improvement could be to aggregate feature embeddings from all networks as input data for each real image, thus synthetically expanding the dataset in a manner that leverages pre-trained feature extraction. Lastly, we explored only one type of classifier. Other classifiers, such as a Random Forest-based method or a deeper classifier, while possibly more prone to overfitting, may also improve performance.

## Methods

### Image acquisition

Deidentified preoperative DICOM image sets for 39 unique patients with histologically confirmed ACP were obtained through the Advancing Treatment for Pediatric Craniopharyngioma consortium (${\hbox {n}}=34$) and the St. Jude Children’s Research Hospital (${\hbox {n}}=5$). Per the Colorado Multiple Institutional Review Board and United States Health and Human Services Regulation 45 CFR 46, this study was exempt from requiring Institutional Review Board approval. Where otherwise concerned, appropriate informed consent was obtained in accordance with the Declaration of Helsinki (v. 2013). Sagittal T1-weighted MRI and axial non-contrast CT image series were selected, based on the fact that the 2 modalities are used in a complementary manner in the clinical setting. A board-certified pediatric neurosurgeon selected individual images from each series, based on their demonstration of the disease process. These were exported as $299\times 299$ pixel JPEG images. This procedure was also performed on analogous imaging studies from 47 unique patients with histologically confirmed non-ACP sellar/suprasellar lesions (NOTACP), which were in the radiological differential diagnosis of ACP. These included pilocytic astrocytoma ($\hbox {n}=12$), germinoma ($\hbox {n}=7$), pilomixoid astrocytoma ($\hbox {n}=6$), optic glioma ($\hbox {n}=4$), pituitary adenoma ($\hbox {n}=3$), arachnoid cyst ($\hbox {n}=3$), prolactinoma ($\hbox {n}=3$), mature teratoma ($\hbox {n}=2$), low grade glioma ($\hbox {n}=2$), renal cell carcinoma ($\hbox {n}=2$), Rathke’s cyst ($\hbox {n}=1$), lipoma ($\hbox {n}=1$), and Langerhans cell histiocytosis ($\hbox {n}=1$). NOTACP image datasets were obtained from the radiology department at Children’s Hospital Colorado ($\hbox {n}=44$) and St. Jude Children’s Research Hospital ($\hbox {n}=3$). For training, we utilized 23 ACP and 30 NOTACP patient datasets. We extracted three representative images per patient and imaging modality (6 images per patient, 318 images total). The test dataset was comprised of 16 ACP and 17 NOTACP patients, with one representative image selected per patient and imaging modality (66 images total; 33 MRI and 33 CT).

### Transfer learning and model architecture

Transfer learning was completed by extracting dense one-dimensional feature vectors (image signatures) using models fully trained on the ILSVRC-2012-CLS dataset. These models are publicly available on TensorFlow Hub (Table 1).

**Table 1:** Pre-trained networks utilized.

Network	Source	Feature vector size
Inception V1	https://tfhub.dev/google/imagenet/inception_v1/feature_vector/1	1024
Inception V2	https://tfhub.dev/google/imagenet/inception_v2/feature_vector/1	1024
Inception V3	https://tfhub.dev/google/imagenet/inception_v3/feature_vector/1	2048
Inception ResNet V2	https://tfhub.dev/google/imagenet/inception_resnet_v2/classification/1	1536
ResNet V1 50	https://tfhub.dev/google/imagenet/resnet_v1_50/feature_vector/1	2048
ResNet V1 101	https://tfhub.dev/google/imagenet/resnet_v1_101/feature_vector/1	2048
ResNet V1 152	https://tfhub.dev/google/imagenet/resnet_v1_152/feature_vector/1	2048
ResNet V2 50	https://tfhub.dev/google/imagenet/resnet_v2_50/feature_vector/1	2048
ResNet V2 101	https://tfhub.dev/google/imagenet/resnet_v2_101/feature_vector/1	2048
ResNet V2 152	https://tfhub.dev/google/imagenet/resnet_v2_152/feature_vector/1	2048
NASNet-A Large	https://tfhub.dev/google/imagenet/nasnet_large/feature_vector/1	4032
PNASNet-5 Large	https://tfhub.dev/google/imagenet/pnasnet_large/feature_vector/2	4320

Modules were accessed using the respective URL and standard TensorFlow Hub methods.

Resultant image signatures were given as inputs to a single fully-connected layer of the standard form

1 $$\begin{aligned} \hat{y} = g(\cdot ) = g(f_{T}(w,x)) = g(wx+b) \end{aligned}$$

where $g(\cdot )$ is the activation function. Prior to activation, input image signatures were transformed via a dropout^5^ layer with feature keep probabilities being one of 25%, 50%, 75%, or 100% (i.e. no dropout). We explored the application of several activation functions (softmax, softplus, softsign, ReLU, leaky ReLU, and log softmax) readily available within the TensorFlow library. Model loss was calculated using the canonical categorical cross-entropy^2,20^ function.

2 $$\begin{aligned} L(y,\hat{y}) = J(w) = -\frac{1}{N}\sum _{n=1}^{N} (y_n \log {\hat{y_n}} + (1 - y_n)\log {(1-\hat{y_n})}) \end{aligned}$$

where applied, model regularization was implemented using $L_{1}$ or $L_{2}$ (Tikhonov) regularization^21,22^ using native TensorFlow commands against either model weights, biases, or both.

Each model architecture was then exposed to one of the following optimizers: Gradient Descent, Adam, Adagrad, Adadelta, RMSProp, Momentum, FTRL, Proximal Adagrad, and Proximal Adadelta^23–30^. Training batch sizes were one of 2, 5, 10, 20, or 120 images. Training duration ranged from 10 to 125 epochs. Lastly, the learning rate utilized for training was one of 0.00001, 0.0001, 0.001, 0.005, 0.01, 0.05, and 0.1.

We acknowledge the F1 score as being a widely utilized accuracy metric for models that are trained on imbalanced datasets, especially in the computer science field. While our original training dataset is indeed slightly imbalanced towards the NOTACP class, we use a balanced test dataset ubiquitously. In addition, our augmented training datasets are numerically balanced. We chose to evaluate network performance using the clinically commonplace metrics of Receiver Operating Characteristic (ROC) curve and area under the ROC curve (AUC) as they more readily translate meaning to clinical practioners^31,32^.

### Genetic algorithm

The genetic algorithm (Algorithm 1) was adapted from a set of publicly available repositories^33,34^, and it is specifically utilized herein as a meta-parameter optimization solution. Briefly, we randomly select one model feature (e.g., loss function, learning rate, batch size, etc.) for each of the features listed in Fig. 2b, this set of features comprises a singular “individual”. For a given generation, we generated 100 of these “individuals”. Each individual is asynchronously processed and the “fitness” of an individual is the AUC value described above. After the full generation has been evaluated, a top fraction is carried over to the next generation. The following generation is created by randomly selecting model features found in the individuals that comprise the top fraction of the the previous generation—akin to “offspring” from a “mother” and “father” set. It is worth noting that genetic algorithms are prone to identifying local minima which can lead to biased optimization results, though we have attempted to mitigate this by using random mutation. A visual schematic for this process can be seen in Fig. 2a.

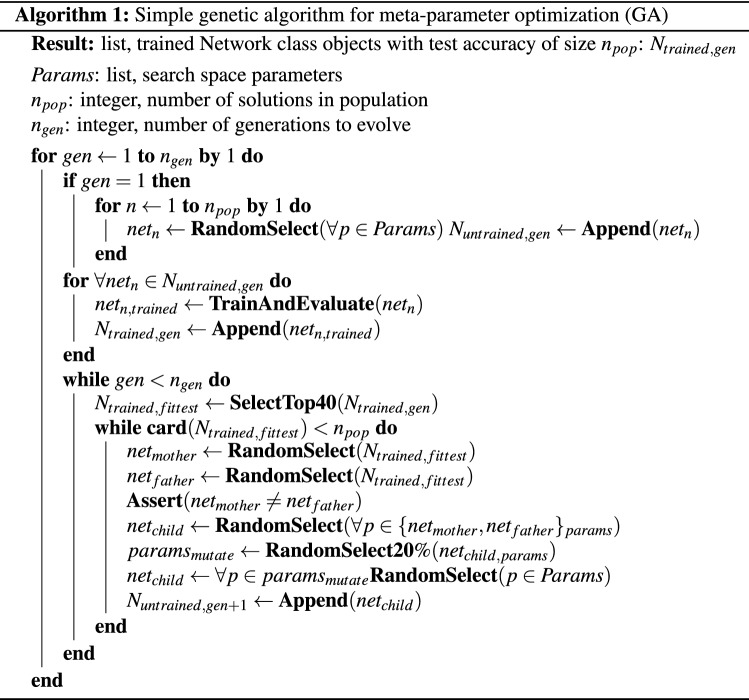



The search space of the genetic algorithm included 19,051,200 possible solutions (12 pre-trained networks $\times 7$ learning rates $\times 3$ batch sizes $\times 5$ training epochs $\times 15$ optimizers $\times 7$ activation functions $\times 4$ dropout rates $\times 4$ regularization methods $\times 3$ training datasets $\times 3$ test datasets). Note, that although there are only 9 optimizers explicitly listed in Fig. 2B that the proximal optimizers have 4 unique variants (no regularization, l1-regularization, l2-regularization, and l1/l2-regularization; as demarcated by the asterisk in the figure), yielding 15 possible optimizers. The AUC “fitness” value is determined for each network by evaluating on the test mentioned above ($\hbox {n}=66$). On our system we were capable of running 10 networks simultaneously at any given time, and runtime for 10 generations with $\hbox {n}=100$ (i.e., 1000 networks) was approximately 1–1.5 days.

### Image augmentation and synthetic data expansion by TANDA

Standard image augmentation was performed using the Augmentor python library^35^. Training data was augmented using a pipeline implementing a random distortion ($\hbox {probability}=0.75$, $\hbox {grid width}=4$, $\hbox {grid height}=4$, $\hbox {magnitude}=8$), followed by a random $90^{\circ }$ rotation ($\hbox {probability}=0.75$), then a random zoom ($\hbox {probability}=0.5$, $\hbox {percentage area}=0.8$), and finally a random left-right flip ($\hbox {probability}=0.5$). CT and MRI data were each sampled using this pipeline for either 100 or 1000 iterations. Test images were sampled using this pipeline, with all probabilities being set to 1.0. Test images were sampled using this pipeline either 10 or 100 times.

Unsupervised GAN-based image generation was performed via minor adaptation to the TANDA python library8 initialized with the following parameters: LSTM-class generator; generator learning rate of $1\times 10^{-4}$; discriminator learning rate of $1\times 10^{-5}$; gamma equal to 0.5; one mean-squared-error (MSE) layer; MSE-term coefficient of $1\times 10^{-3}$; transformation sequence length of 10; no per-image standardization; trained using a batch size of 5 and for a duration of 5 epochs. We sought to extract the generated images as JPEG files for visualization, as opposed to direct import into an end-classifier. 1000 ACP and 1000 NOTACP synthetic images were generated for both CT and MRI modalities.

### Computational hardware and software

All computational programs were performed on a 64-bit RedHat Enterprise Linux HPC running CentOS 7.4.1708. Python based programs were executed in a virtual environment containing Python 3.6 with the following modules: Augmentor (v 0.2.2)^35^, Matplotlib (v 2.2.2)^36^, Numpy (v 1.14.15)^37^, Pandas (v 0.23.3)^38^, Ray (v 0.6.4)^39^, Sci-kit Image (v 0.14.0)^40^, TensorFlow (v 1.12.0)^11^, and TensorFlow Hub (v 0.2.0)^11^.

## Data Availability

The dataset analyzed during the current study is available from the corresponding author on reasonable request.

## References

[1] LeCun Y, Bengio Y, Hinton G (2015). Deep learning. Nature.

[2] Goodfellow I, Bengio Y, Courville A (2016). Deep Learning.

[3] Gulshan V (2016). Development and validation of a deep learning algorithm for detection of diabetic retinopathy in retinal fundus photographs. JAMA.

[4] Russakovsky, O. *et al.* Imagenet large scale visual recognition challenge. *CoRR* arXiv:abs/1409.0575 (2014).

[5] Srivastava N, Hinton G, Krizhevsky A, Sutskever I, Salakhutdinov R (2014). Dropout: a simple way to prevent neural networks from overfitting. J. Mach. Learn. Res..

[6] Lu J (2015). Transfer learning using computational intelligence: a survey. Knowl. Based Syst..

[7] Bengio, Y., Courville, A. C. & Vincent, P. Unsupervised feature learning and deep learning: a review and new perspectives. *CoRR* arXiv:abs/1206.5538 (2012).

[8] Ratner, A. J., Ehrenberg, H. R., Hussain, Z., Dunnmon, J. & Ré, C. Learning to compose domain-specific transformations for data augmentation. arXiv:1709.01643 (2017). PMC5786274 29375240

[9] Gupta P, Jalali R (2017). Long-term survivors of childhood brain tumors: impact on general health and quality of life. Curr. Neurol. Neurosci. Rep..

[10] Norris GA (2019). Diagnostic accuracy of neuroimaging in pediatric optic chiasm/sellar/suprasellar tumors. Pediatr. Blood Cancer.

[11] Abadi, M. *et al.* Tensorflow: large-scale machine learning on heterogeneous distributed systems. *CoRR* arXiv:abs/1603.04467 (2016).

[12] He, K., Zhang, X., Ren, S. & Sun, J. Deep residual learning for image recognition. CoRR arXiv:abs/1512.03385 (2015).

[13] Ioffe, S. & Szegedy, C. Batch normalization: Accelerating deep network training by reducing internal covariate shift. CoRR arXiv:abs/1502.03167 (2015).

[14] Liu, C. *et al.* Progressive neural architecture search. CoRR arXiv:abs/1712.00559 (2017).

[15] Szegedy, C., Ioffe, S. & Vanhoucke, V. Inception-v4, inception-resnet and the impact of residual connections on learning. CoRR arXiv:abs/1602.07261 (2016).

[16] He, K., Zhang, X., Ren, S. & Sun, J. Identity mappings in deep residual networks. CoRR arXiv:abs/1603.05027 (2016).

[17] Szegedy, C., Vanhoucke, V., Ioffe, S., Shlens, J. & Wojna, Z. Rethinking the inception architecture for computer vision. CoRR arXiv:abs/1512.00567 (2015).

[18] Perez, L. & Wang, J. The effectiveness of data augmentation in image classification using deep learning. CoRR arXiv:abs/1712.04621 (2017).

[19] Ciresan, D. C., Meier, U., Gambardella, L. M. & Schmidhuber, J. Deep big simple neural nets excel on handwritten digit recognition. CoRR arXiv:abs/1003.0358 (2010). 10.1162/NECO_a_00052 20858131

[20] Murphy KP (2013). Machine Learning: A Probabilistic Perspective.

[21] Ng, A. Y. Feature selection, l1 vs. l2 regularization, and rotational invariance. In *Proceedings of the Twenty-First International Conference on Machine Learning*, ICML ’04, 78, 10.1145/1015330.1015435 (Association for Computing Machinery, New York, NY, USA, 2004).

[22] Regularization for sparsity: L1 regularization. https://developers.google.com/machine-learning/crash-course/regularization-for-sparsity/l1-regularization. Accessed 15 June 2020

[23] Kingma, D. P. & Ba, J. Adam: a method for stochastic optimization (2014). Cite arxiv:1412.6980Comment: Published as a conference paper at the 3rd International Conference for Learning Representations, San Diego, 2015.

[24] Cauchy MA (1847). Methode generale pour la resolution des systemes d’equations simultanees. C. R. des seances de l'academie des Sci..

[25] Duchi J, Hazan E, Singer Y (2011). Adaptive subgradient methods for online learning and stochastic optimization. J. Mach. Learn. Res..

[26] Duchi, J. & Singer, Y. Proximal and first-order methods for convex optimization. https://ppasupat.github.io/a9online/uploads/proximal_notes. Accessed 15 June 2020

[27] Zeiler, M. D. ADADELTA: an adaptive learning rate method. CoRR arXiv:abs/1212.5701 (2012).

[28] McMahan, H. B. *et al.* Ad click prediction: a view from the trenches. In *Proceedings of the 19th ACM SIGKDD International Conference on Knowledge Discovery and Data Mining*, KDD ’13, 1222–1230, 10.1145/2487575.2488200 (Association for Computing Machinery, New York, NY, USA, 2013).

[29] Sutskever, I., Martens, J., Dahl, G. & Hinton, G. On the importance of initialization and momentum in deep learning. In *Proceedings of the 30th International Conference on International Conference on Machine Learning - Volume 28*, ICML’13, III–1139–III–1147 (JMLR.org, 2013).

[30] Singer Y, Duchi JC, Bengio Y, Schuurmans D, Lafferty JD, Williams CKI, Culotta A (2009). Efficient learning using forward-backward splitting. Advances in Neural Information Processing Systems.

[31] Hanley JA, McNeil BJ (1982). The meaning and use of the area under a receiver operating characteristic (roc) curve. Radiology.

[32] Bradley AP (1997). The use of the area under the roc curve in the evaluation of machine learning algorithms. Pattern Recognit..

[33] Harvey, M. Let’s evolve a neural network with a genetic algorithm. https://blog.coast.ai/lets-evolve-a-neural-network-with-a-genetic-algorithm-code-included-8809bece164. Accessed 15 June 2020.

[34] Larson, W. Genetic algorithms: cool name and damn simple. https://lethain.com/genetic-algorithms-cool-name-damn-simple/. Accessed 15 June 2020.

[35] Bloice, M. D., Stocker, C. & Holzinger, A. Augmentor: an image augmentation library for machine learning. CoRR arXiv:abs/1708.04680 (2017).

[36] Hunter JD (2007). Matplotlib: A 2d graphics environment. Comput. Sci. Eng..

[37] Oliphant TE (2007). Python for scientific computing. Comput. Sci. Eng..

[38] McKinney, W. *et al.* Data structures for statistical computing in python. In *Proceedings of the 9th Python in Science Conference*, vol. 445, 51–56 (Austin, TX, 2010).

[39] Moritz, P. *et al.* Ray: a distributed framework for emerging AI applications. CoRR arXiv:abs/1712.05889 (2017).

[40] van der Walt S (2014). scikit-image: image processing in Python. PeerJ.

